# Iron overload and iron chelating agent exposure in anemia-associated outer retinal degeneration: a case report and review of the literature

**DOI:** 10.1186/s12886-021-02030-1

**Published:** 2021-07-13

**Authors:** Mohamed Belmouhand, Christina Eckmann-Hansen, Tomas Ilginis, Eva Birgitte Leinøe, Bo Kok Mortensen, Michael Larsen

**Affiliations:** 1 Department of Ophthalmology, Rigshospitalet, Copenhagen University Hospital, Glostrup, Denmark; 2 Department of Clinical Medicine, Faculty of Healthy and Medical Science, University of Copenhagen, Copenhagen, Denmark; 3 Department of Hematology, Rigshospitalet, Copenhagen University Hospital, Copenhagen, Denmark; 4 Department of Hematology, Herlev and Gentofte Hospital, Copenhagen University Hospital, Herlev, Denmark

**Keywords:** Anemia, Iron chelation, Iron overload, Deferoxamine, Retinopathy, Case report

## Abstract

**Background:**

Deferoxamine retinopathy is the informally designated term used to describe a characteristic pattern of outer retinal degeneration in iron-overloaded chronic anemia patients who are treated with deferoxamine. We hypothesize that insufficiently treated iron overloading and not only deferoxamine is the cause of the retinal degeneration. Our case report is based on exposure histories of two anemia patients and literature review.

**Case presentation:**

Both anemia patients presented with bilateral visual loss secondary to photoreceptor and retinal pigment epithelium degeneration. Chart review showed that visual loss came after a year-long slow, and rather monotonous rise in plasma ferritin concentrations, with no obvious relation to iron chelator exposure. In one patient, the onset of symptomatic visual loss came after a bout of fever followed by two additional febrile episodes, all accompanied by plasma ferritin spikes. Adjustment of iron chelation therapy did not improve visual function. Experimental studies clearly show that both systemic and intraocular exposure to iron ions can induce retinal degeneration.

**Conclusion:**

The available evidence indicates that retinal degeneration in chronic anemia patients treated by deferoxamine is cause by insufficient iron chelation, not by deferoxamine. The actual role of iron chelating agents may be to promote a long enough survival to allow the slow development of retinal siderosis.

## Introduction

The iron content of the human body is regulated at the level of intestinal absorption. There is no regulated form of excretion in individuals with excessive iron store. This can lead to iron toxicity in patients with a chronic need for blood transfusions. If untreated, many patients with hematological disease will therefore die from heart and liver failure within 10 years [1]. To avoid fatal cardiac complications of hemosiderosis (systemic iron overload), individuals with chronic transfusion-treated anemia are therefore treated with an iron chelating agent.

The introduction of the iron chelating agent deferoxamine in 1968 was followed by a marked reduction in mortality from hemosiderosis. Subsequently, the term deferoxamine retinopathy was coined for a characteristic pigmented degeneration of the outer retina with annular visual defects and night blindness that was seen among chronic anemia patients treated with an iron chelating agent (e.g. deferoxamine) [2, 3]. Several studies, primarily case-reports, have suggested that iron chelators may cause retinopathy [3–7]. However, other studies have presented results that contradict this notion [8, 9], and the demonstration of a potential role of trace element imbalance in optic nerve degeneration further complicates the evaluation of visual loss in patients suffering from chronic anemia [10]. Finally, the potential role of iron in inducing retinal degeneration has received little attention in the context of chronic anemia management.

Human retinal pigment epithelium (RPE) cells contain transferrin receptors that enable endocytosis-mediated iron uptake from the choroid, and iron is known to be toxic to the retina, especially the RPE [11, 12]. The degenerative damage is believed to originate from iron-induced oxidative stress [12]. We therefore hypothesize that prolonged hemosiderosis plays a major role in the development of retinopathy in transfusion-dependent anemia patients, alone or in combination with iron chelating agents.

The aim of the study was to examine iron levels and deferoxamine treatment in two patients with chronic anemia before, during and after vision loss to more accurately determine the role of hemosiderosis in what is commonly called deferoxamine retinopathy. We have reviewed the evidence base of this nosological entity in the light of data from two patients with chronic anemia who consented to having their data published.

## Case presentation

### Patient 1

**Fig. 1:**
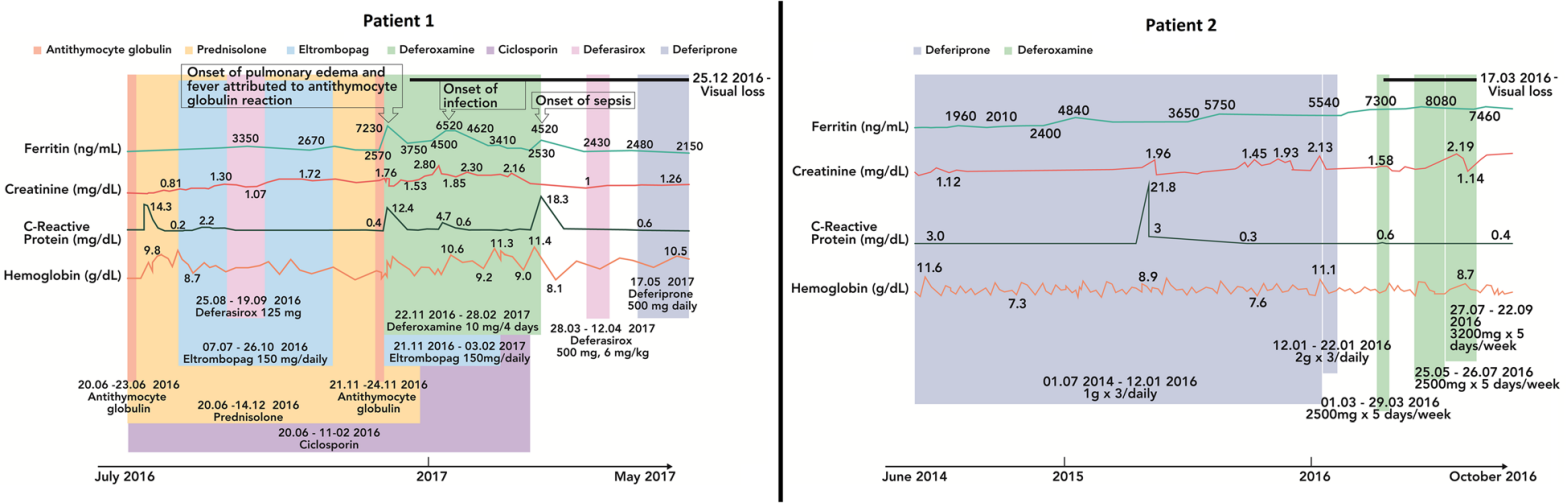
Timeline chart for patient 1 and 2 with relevant blood tests and drug exposure. Legend: Patient 1, timeline from 4 years after presentation with chronic anemia through half a year leading up to the onset of visual loss and 4 months thereafter, showing the exposure to cyclosporin, antithymocyte globulin, iron chelators (deferasirox, deferoxamine and deferiprone), thrombopoietin analog (eltrombopag), onset and persistence of visual loss, onset of fever and pulmonary edema, and plasma concentrations of ferritin, creatinine, c-reactive protein and hemoglobin. Additionally, the patient received blood transfusions throughout the period of observation. Patient 2, timeline from 3 years after the diagnosis of blood dyscrasia through 37 months of follow-up. Deferiprone was exchanges for deferoxamine due to deviating renal function and inefficiency. Additionally, the patient received blood transfusions throughout the period of observation. SI conversion factor: Ferritin, ng/mL ➔ μg/L, multiply by 1; creatinine, mg/dL ➔ μmol/L, multiply by 88.42; c-reactive protein, mg/dL ➔ mg/L, multiply by 10; and hemoglobin, g/dL ➔ mmol/L, multiply by 0.62

**Fig. 2:**
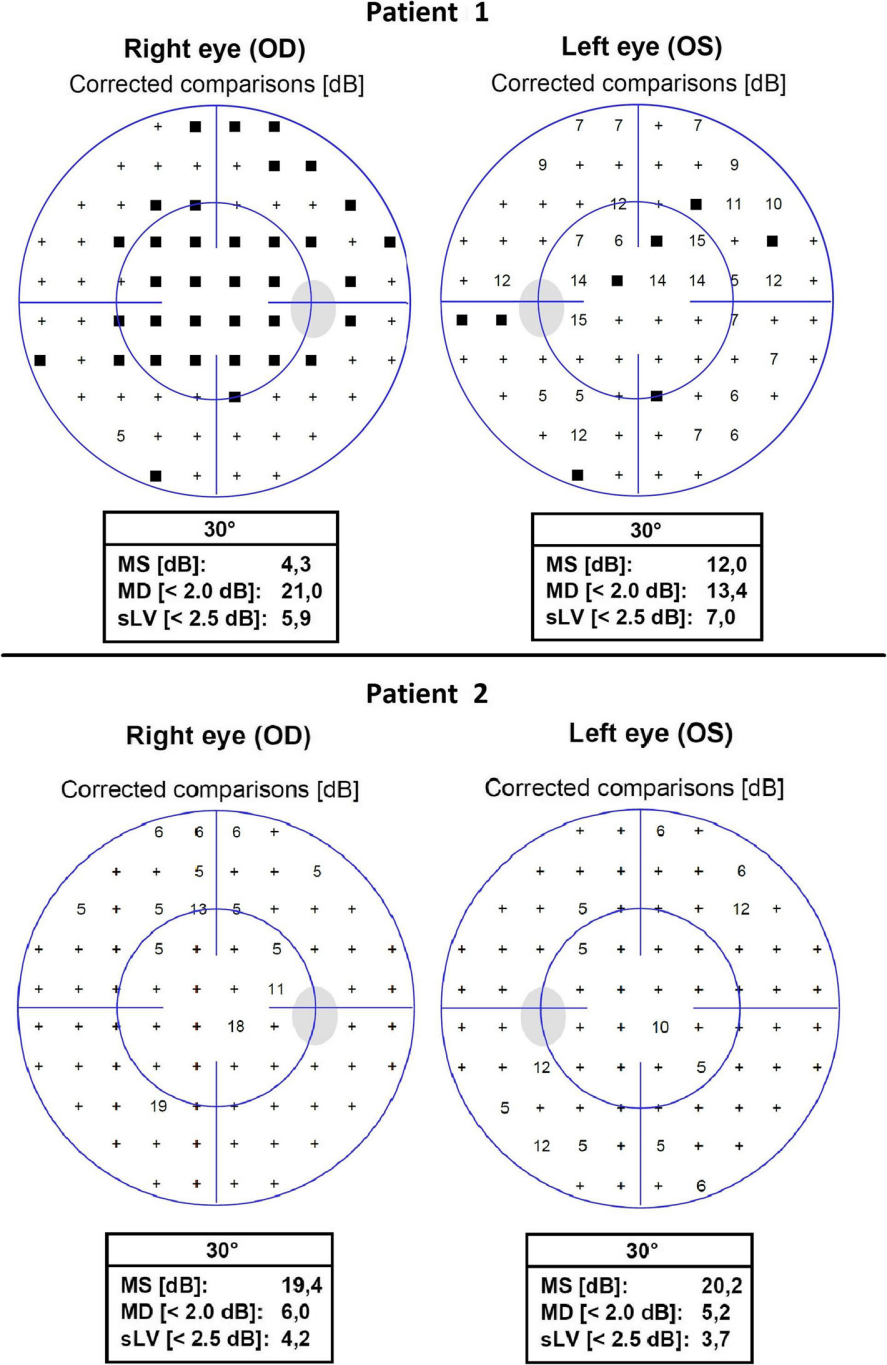
Automated perimetry examination of patient 1 and 2. Legend: Automated perimetry, 30-2, (Octopus 900, Haag-Streit, Switzerland) in two patients, patient 1 above at presentation and patient 2 below at 2 years after presentation. MS, mean sensitivity; MD, mean defect; and sLV, square root of loss variance

**Fig. 3:**
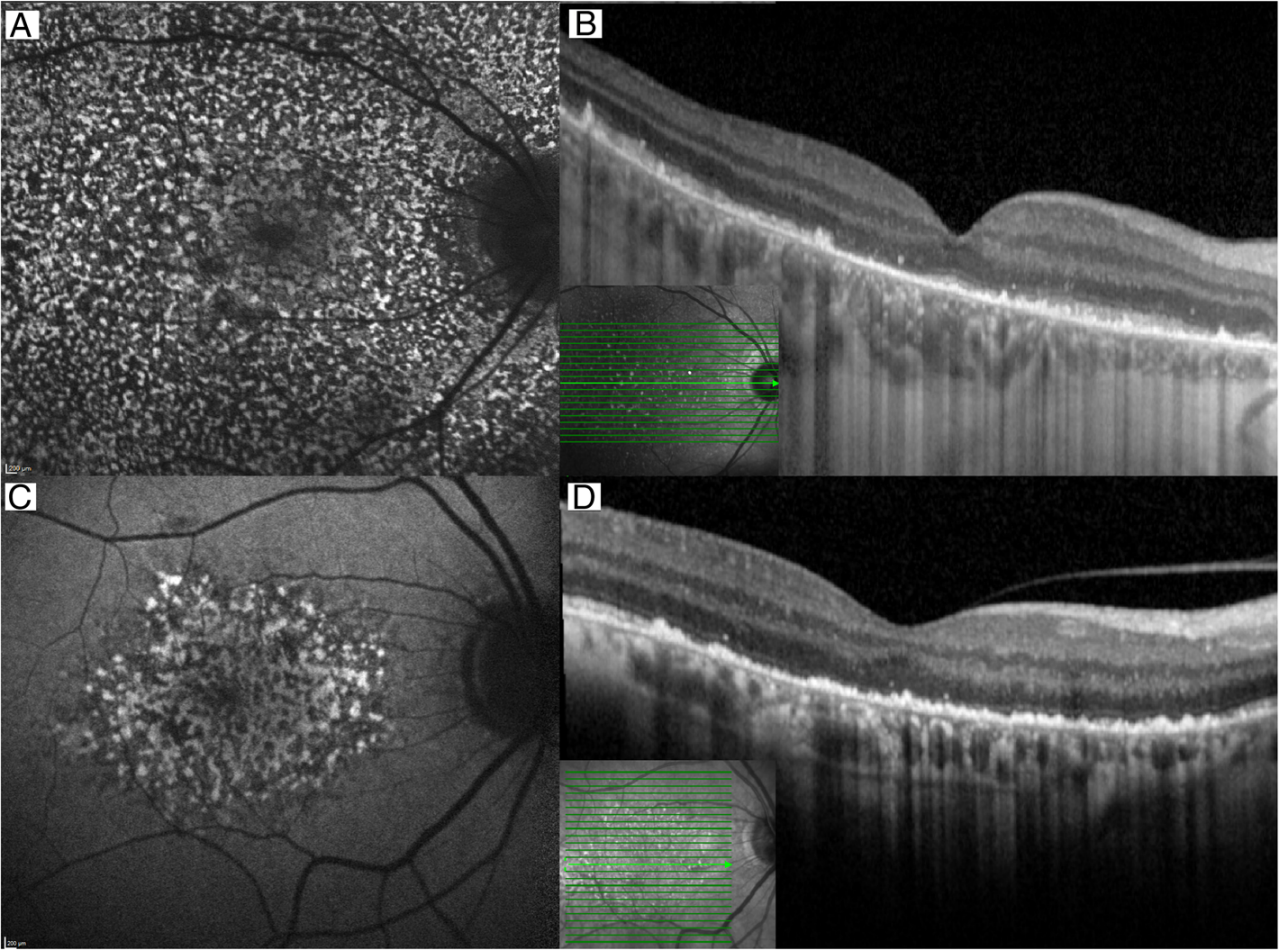
Optical coherence tomography and blue light fundus autofluorescence imaging of retinal defects. Legend: In patient 1 (A + B), a diffuse dotted blue light fundus autofluorescence of the right eye shows widespread defects in the whole macula (**A**). The corresponding horizontal transfoveal optical coherence tomography shows degeneration and irregularities in the outer retina (**B**). In patient 2 (C + D), a more localized defect is visible in the parafoveal region on blue light fundus autofluorescence (**C**). The optical coherence tomography shows the same outer retinal defects as in case 1 but temporally an intact outer retina is seen (**D**). In case 1 the images were acquired during initial visit, and in case 2 the images were acquired at follow-up 6 months after initial visit. Scans were acquired on Spectralis OCT2 (Heidelberg Engineering, Heidelberg, Germany)

**Fig. 4:**
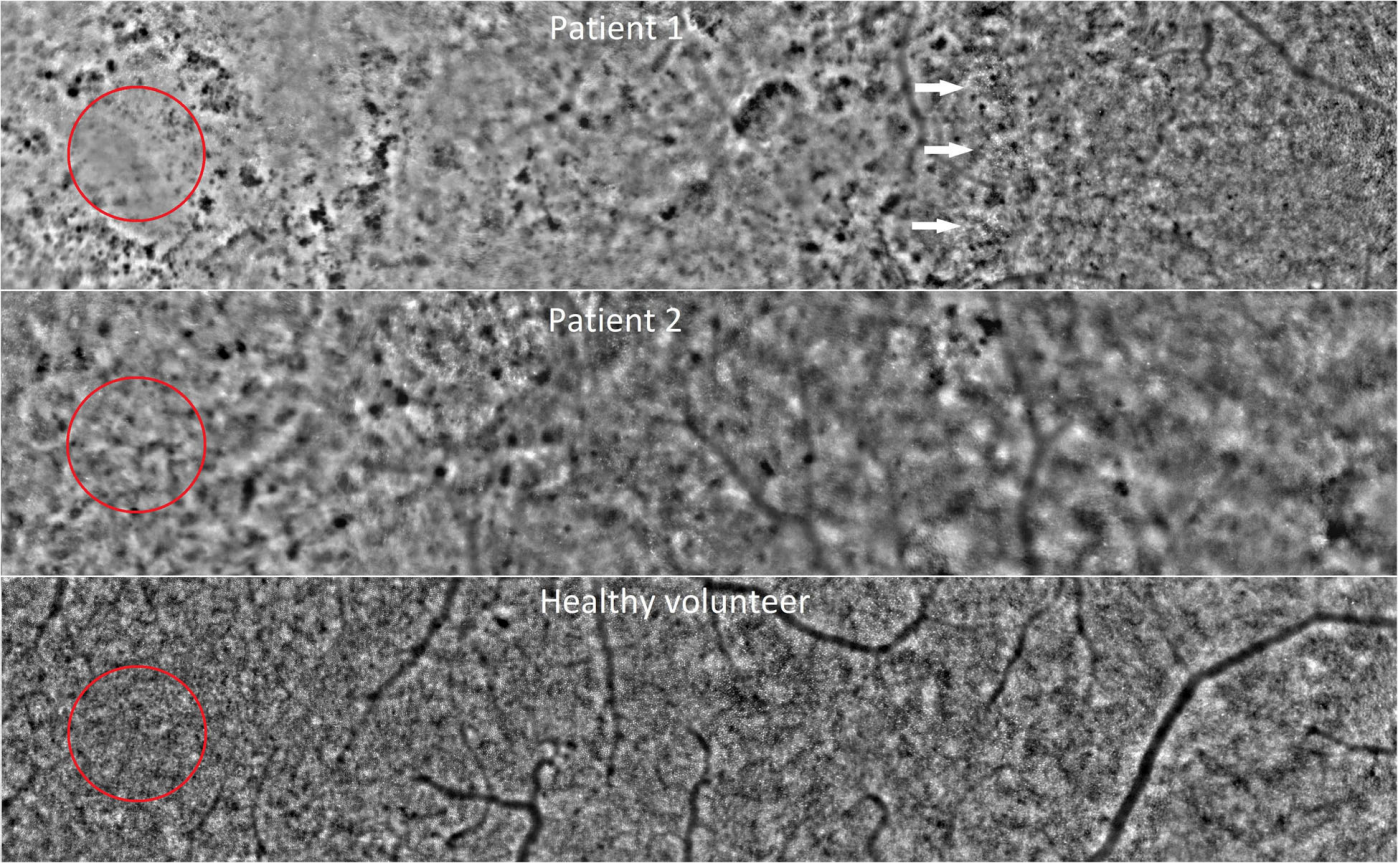
Adaptive optics fundus photography of photoreceptor mosaic. Legend: Patient 1 (**A**), patient 2 (**B**) and a healthy age-matched volunteer (**C**), spanning from the fovea (indicated by red circles) to a position 14 degrees temporal of the foveal center. Patient 1 is remarkable for having a transitional zone (white arrows) between injured granular retina on the foveal side and more normal retina on the temporal side. Patient 2 shows patchy absence or attenuation of the photoreceptor matrix. The healthy volunteer had a normal photoreceptor distribution. The images of patient 1 and 2 were recorded 5 and 4 months, respectively, after presentation. Acquired on RTX-1 (Imagine Eyes, Orly, France) on flood-illuminated adaptive optics fundus photography

A 72-year-old man presented with blurred vision in both eyes beginning 1 week earlier as a dark, partially transparent area in the center of both visual fields. The dark area gradually expanded and changed to a pericentral dark ring in both eyes. He had suffered from aplastic anemia for 4 years and received one blood transfusion per week for 8 months when he presented. He had also received five infusions of 301 mg antithymocyte globulin 1 month before the onset of visual loss (Fig. 1) and oral deferasirox beginning 4 months earlier. Deferasirox was discontinued after 1 month due to renal impairment and changed to deferoxamine infusion 1 month prior to visual loss. Past medical history included appendectomy (21 y), encephalitis (42 y), bilateral cataract surgery with pseudophakia (51 y) and curative treatment of prostate cancer (69 y). Best-corrected Snellen visual acuity (BCVA) was 0.6 in the right eye and 0.9 in the left eye, a serous detachment of the macula was found in both eyes and applanation tonometry was normal. Visual acuity deteriorated to 0.3 in both eyes 3 months later. A presumptive diagnosis of deferoxamine retinopathy prompted a switch to oral deferiprone, but visual acuity decreased to worse than 0.1 in both eyes over the following year. From half a year before presentation to half a year after presentation, plasma ferritin – a surrogate measure of iron stores – had ranged from 2000 to 7230 ng/mL (SI: 2000 to 7230 μg/L), reference range 15–320 ng/mL, with prominent peaks immediately before and after the onset of visual loss (Fig. 1). Patchy visual field sensitivity loss (Fig. 2) was seen corresponding to hyper- and hypofluorescent degeneration of the outer retina in both maculae (Fig. 3) and severely reduced cone photoreceptor density out 14 degrees from the foveola (Fig. 4). There were no other signs of hemosiderosis.

### Patient 2

A 70-year-old male erythropoiesis-deficient anemia patient with floaters, flashes and poor color vision was seen with BCVA 0.9 in the right eye and 1.0 in the left eye. He had previously been diagnosed with type 2 diabetes mellitus (57 y), hypertension (57 y), diabetic nephropathy (68 y) and myelodysplastic syndrome with ring sideroblasts (65 y). Blood transfusions had been administered from the past 2 years prior and was continued after the onset of visual symptoms. Deferiprone, administered until 8 months earlier, had been switched to deferoxamine due to renal impairment (Fig. 1). A tentative diagnosis of vitreous degeneration was made, but 6 months later BCVA had dropped to 0.2 in both eyes. The suspicion that deferoxamine was the cause prompted discontinuation of this drug without chelator substitution. One year later BCVA had increased to 0.8 in the right eye and 0.4 in the left eye. It then fell to 0.4 in both eyes when seen 55 months after presentation, when geographic atrophy was seen in the left macula. Over the 6 years up to his most recent visit, plasma ferritin had ranged from 1500 to 10,030 ng/mL, with a peak near the onset of visual loss (Fig. 1). Visual field sensitivity was moderately diffusely reduced (Fig. 2) and both maculae showed granular hyper- and hypoautofluorescence with corresponding photoreceptor and pigment epithelium atrophy (Figs. 3 and 4).

## Discussion and conclusions

The outer retinal degeneration seen in our two patients fits the description of what is called deferoxamine retinopathy. It is remarkable, however, that neither case showed a clear temporal association between iron chelator exposure, disease duration or disease severity, and retinal degeneration. Remarkably, the onset of subjective visual loss occurred on the chronically rising slopes of plasma ferritin concentration curves that had exceeded the recommended maximum of 1000 ng/mL for years [13]. While this maximum has been set to safeguard the heart, there is insufficient data to estimate the level of ferritin that is safe for the retina. We did not observe any uniform pattern of changes in retinal function or structure and fluctuations in hemoglobin or c-reactive protein.

Competing with the notion that deferoxamine in itself should be retinotoxic [4, 14], there is evidence that iron overload can induce outer retinal degeneration by promoting the production of reactive oxygen species and an inherent increase in oxidative stress [12, 15]. Independent of the blood-retina barrier, human RPE cells have an abundance of transferrin receptors which permit endocytosis-mediated iron uptake from the choroid [11]. Of particular note, our patients’ ocular characteristics are comparable those seen in hemochromatosis [16], in experimental intraocular iron toxicity [17, 18] and in intraocular ferrous foreign body retention, where the functional deficit is partially reversible [19], as was the visual loss in our patient 2.

The mechanism or etiology of a possible deferoxamine-induced toxicity is not understood; however, it is plausible that deferoxamine affects iron mobilization locally in the retina and thereby causes atrophy. Furthermore, clinical cases of presumed deferoxamine retinopathy have all occurred in patients with iron overload during some period of their disease [3], and rabbits with intraocular iron foreign bodies are protected from retinal degeneration by deferoxamine [20]. Deferiprone, an oral iron chelator, was shown to protect against retinal degeneration induced by systemic iron overload in mice [21, 22]. While deferoxamine can depress the electroretinogram, the effect is transient and was observed in patients with concurrent hemosiderosis [3].

Preclinical data from animal studies shows that iron overload can induce retinal damage [17, 23–25]. In addition, iron overload can also accelerate cell death in rats exposed to partial nerve head crush [26]. Knock-out of TIM2, the receptor for H-ferritin in Müller cells of the mouse retina, has led to iron overload and consequently production of reactive oxygen species and subsequent retinal degeneration [27]. Salvianic acid A, an extract from Chinese herbs, and Puerarin are agents used in alternative medicine that have been shown to ameliorate iron overload-induced toxicity in mice [28, 29]. The positive actions of these two drugs are believed to be through regulation of iron-handling proteins that possibly aid in iron chelation and attenuation of oxidative stress. These two drugs have not been tested in humans with hemosiderosis.

Ferroptosis, a recently described cell death mechanism that dependents on an abundant concentration of iron [30], may theoretically be involved in retinal damage by promoting retinal cell death in hemosiderosis. Thus, a study has found evidence that ferroptosis contributed to cell death in cultured human RPE cells exposed to iron overload, and that deferoxamine attenuated the rate of cell death [31].

Only a speculative case association with retinal degeneration has been reported for deferasirox and none for deferiprone, which are two alternative clinical iron chelating drugs [32]. Thus, the analysis of clinical cases may potentially be confounded by interaction between a reversible functional effect of deferoxamine and a more permanent effect of iron on the retina [3]. Notably, most clinical reports linking deferoxamine with retinal degeneration have not accounted for ferritin levels prior to, during or after vision loss. We therefore suspect that ferritin spikes or burst of cytokine release from disintegrating T-cells, as seen during antithymocyte therapy [33], may promote retinal degeneration, as may the natural accumulation of iron in the retina with aging [34]. Whether antithymocyte therapy or other unknown dysfunction in iron metabolism caused the retinal damage in our patient no. 1 to become irreversible can only be hypothesized.

Our two cases expand OCT-based demonstrations of attenuation and irregularity of the photoreceptor and RPE layers in the macula [5, 14]. Cone photoreceptor counts in affected areas were about half of what is normal, with considerable focal variations confined to the area within the temporal vascular arcades.

The complexity of the typical clinical course in anemia limits the retrospective analysis of causality in retinopathy cases, of whom the ones with the highest iron load are the ones who received the most intensive chelation therapy. Although our two cases were highly valuable in understanding the complexity of chronic anemia and retinopathy, we cannot generalize or establish cause-effect relationships based on our findings.

In conclusion, retinal injury in iron-overload anemia should not unreservedly be attributed to iron chelation therapy. Insufficient iron chelation should be considered as an alternative etiology. Proactive eye examination, meticulous clinical documentation and awareness of the guideline upper plasma ferritin level of 1000 ng/mL may help enable rational analysis of cases and reduction of the incidence of retinal degeneration in chronic anemia patients.

## Data Availability

All data generated or analyzed during this study are included in this published article.

## Abbreviations

BCVA: Best-corrected visual acuity
RPE: Retinal pigment epithelium
